# Nuclear Gene Variation in Wild Brown Rats

**DOI:** 10.1534/g3.112.004713

**Published:** 2012-12-01

**Authors:** Rob W. Ness, Yao-Hua Zhang, Lin Cong, Yu Wang, Jian-Xu Zhang, Peter D. Keightley

**Affiliations:** * Institute of Evolutionary Biology, University of Edinburgh, Edinburgh, EH9 3JT, United Kingdom; † State Key Laboratory for Integrated Pest Management, Institute of Zoology, Chinese Academy of Sciences, Bei Chen Xi Lu 1, Beijing 100101, China, and; ‡ Institute of Plant Protection, Heilongjiang Academy of Agricultural Sciences, Harbin 150086, China

## Abstract

Although the brown rat (*Rattus norvegicus*) is widely used as a model mammal throughout biological sciences, little is known about genetic variation in wild rat populations or the relationship of commonly used inbred strains to their wild relatives. We sampled wild brown rats from the species’ presumed ancestral range in NW China and from a derived population in the UK and estimated nucleotide diversity and population subdivision, based on the sequences of 30 autosomal protein-coding loci. Neutral genetic diversity was close to 0.2% in both populations, which is about five times lower than diversity at the orthologous sites in a population of wild house mice from the species’ putative ancestral range in India. We found significant population differentiation between UK and Chinese populations, as assessed by *F_st_* and the program STRUCTURE. Based on synonymous diversity and divergence between the brown rat and house mouse, we estimate that the recent effective population size in brown rats is approximately 130,000 (approximate 95% confidence interval 85,000-184,000), about fivefold lower than wild house mice.

The brown rat is the leading animal model in physiology and pharmacology research, and, after the house mouse, is the most widely studied model mammal in genetics. Yet, little is known about the origin of inbred rat strains or the genetic relationship between inbred strains and wild rats. Studies of the mitochondrial genome (Brown and Simpson 1981; Li *et al.* 1999; Lin *et al.* 2012), allozymes (Bender *et al.* 1985; Cramer *et al.* 1988), and random amplified polymorphic DNA markers (Jiang *et al.* 2005) hint at the presence of substantial genetic diversity in wild brown rat populations. Geographic variation for morphological traits in Chinese populations has been reported (Wu 1982), and four subspecies have been recognized on the basis of variation in morphology (Wu 1982; Wilson and Reeder 2005). However, the amount of genetic variation in nuclear genes and the extent of geographical differentiation among populations in nature are not known.

There is substantially more information on genetic diversity among inbred laboratory rat strains. Surveys of microsatellites (Canzian 1997; Thomas *et al.* 2003) and single nucleotide polymorphisms [SNPs (Smits *et al.* 2004; STAR Consortium 2008)] suggest that inbred strains are genetically diverse and distinct from Brown Norway strains, which are assumed to be the most closely related strains to wild brown rats (Thomas *et al.* 2003). Brown Norway is the reference strain for the rat genome project (Gibbs *et al.* 2004). *Rattus* species most likely originated in Southeast Asia, which is the center of current rat species diversity (Rowe *et al.* 2011). *Rattus norvegicus* is assumed to have evolved on the plains of Asia in NW China and Mongolia, where wild brown rats are still found in what is presumed to be their native habitat. Although the black rat (*Ratrrus rattus*) is known from antiquity in Europe (Barnett 2001), *R. norvegicus* is believed to have reached Europe much later, probably between the 16th and 18th centuries, from where it has spread worldwide and largely displaced *R. rattus* in temperate regions.

In contrast to wild rats, there is a good deal of information on the genetic diversity in wild house mice (*Mus musculus*). House mouse subspecies vary in their mean nucleotide diversity (Baines and Harr 2007; Salcedo *et al.* 2007) and the extent of gene flow between the different subspecies varies across the genome (Teeter *et al.* 2008). Nuclear gene diversity is greatest in populations from their putative ancestral ranges, which are Iran and NW India in the cases of *M. m. domesticus* and *M. m. castaneus*, respectively (Baines and Harr 2007). NW India is also believed to be the ancestral range of the species complex as a whole (Din *et al.* 1996). Diversity from the putative ancestral range of *M. m. musculus* (NW Afghanistan) is currently unknown (Baines and Harr 2007). Synonymous site diversity of protein-coding genes in *M. m. castaneus and M. m. domesticus* ancestral populations is more than eightfold and threefold greater, respectively, than observed in human populations [(Baines and Harr 2007; Halligan *et al.* 2010) D. L. Halligan, A. Kousathanas, R. W. Ness, B. Harr, L. Eöry, H. Li, T. M. Keane, D. J. Adams, and P. D. Keightley, unpublished results], presumably a consequence of a substantially higher effective population sizes in wild mice. We therefore expected to see a similar pattern in brown rats, with diversity greatest in individuals from the ancestral range, and overall diversity levels comparable with mice, under the expectation that the rat ancestral effective population size is likely to have been very large. Here, we report the first survey of nuclear gene sequence diversity in wild brown rats. We sequenced 30 autosomal loci in a sample of individuals from the species’ presumed ancestral range in NW China and from a derived population in the UK.

## Materials and Methods

We trapped 22 *R. norvegicus* in a ∼500-km^2^ area around the city of Harbin, Heilongjiang Province, China in 2011 (supporting information, Table S1). We avoided sampling closely related individuals by ensuring that trapping locations were a minimum of 100 m apart. We also obtained 7 *R. norvegicus* from a derived population in the UK from seven sites in 2012 (Table S1). We applied Sanger sequencing of both DNA strands to obtain the partial sequences of 30 protein-coding loci (Table S2). Sequences were assembled using Sequencher 4.7, and ambiguous and variant sites were confirmed visually using both strands. We compared patterns of nucleotide polymorphism at amino acid replacement and synonymous sites between the two rat populations and between rat and mouse using polymorphism data from orthologous loci from 10 wild *Mus m. castaneus* from NW India (D. L. Halligan, A. Kousathanas, R. W. Ness, B. Harr, L. Eöry, H. Li, T. M. Keane, D. J. Adams, and P. D. Keightley, unpublished results).

## Results

### Nucleotide variation in wild rats

Table 1 compares two measures of nucleotide variation, nucleotide diversity, *θ_π_* (Tajima 1983), and nucleotide polymorphism, *θ_W_* (Watterson 1975). The most striking feature of the results is the relatively low level of nucleotide diversity in each population. Synonymous diversity in both populations is ∼0.2%, which is at the low end of the range for known vertebrate species (Lynch 2007), and only twice what is typically observed in humans, which have very low genetic diversity (Li and Sadler 1991; Cargill *et al.* 1999). On the other hand, diversity in orthologous loci of *M. m. castaneus* is 5.2-fold greater than in wild rats. Diversity in the UK rat population is marginally lower than diversity in rats from the Chinese population, but the difference is nonsignificant (χ^2^,1 df = 1.8, *P* = 0.18). Combining all 13,408 sites at which we have sequence data, there were no fixed differences, 18 polymorphisms shared between the two populations,17 private to the UK, and 42 private to China. *θ_W_* is generally greater than *θ_π_*, suggesting an excess of rare alleles compared with the neutral expectation, which is reflected in a marginally negative Tajima’s *D* across all loci for both the Chinese (*D* = −0.36) and UK rats (*D* = −0.16) and wild rats combined (*D* = −0.61). To determine whether there is evidence for a departure from neutrality in any of the 30 loci across two populations, we estimated Tajima’s *D* for each locus in each population, and we conducted 1000 coalescent simulations to estimate the null distribution. There is little evidence for departures from neutrality, because only 5 of 60 values differed significantly from the neutral expectation (*P* < 0.05) and in each of these loci there is only one or two synonymous sites segregating. In addition, analysis of the genotype frequencies in the two populations showed no consistent departure from Hardy-Weinberg expectation nor did the McDonald-Kreitman test (McDonald and Kreitman 1991) detect any signature of positive selection (data not shown).

**Table 1:** Summary of nucleotide polymorphism at replacement and synonymous sites

	Replacement Polymorphism			Synonymous Polymorphism		
Population (No. Alleles)	No. Sites (No. Variable)	*θ_π_*	*θ_W_*	No. Sites (No. Variable)	*θ_π_*	*θ_W_*
China (44)	10035 (19)	0.00037	0.00044	2910 (31)	0.00216	0.00245
UK (14)	9911 (14)	0.00041	0.00045	2886 (12)	0.00163	0.00132
All rats (58)	9970 (22)	0.00038	0.00048	2893 (29)	0.00215	0.00217
*M*. *m. castaneus* (20)	16512 (153)	0.00199	0.00263	4784 (226)	0.0112	0.0134

Included are the values for the Chinese and UK populations, as well as their combined values. Values from the orthologous sequences of 10 wild caught *Mus musculus castaneus* are provided for comparison.

### Population structure

**Figure 1:**
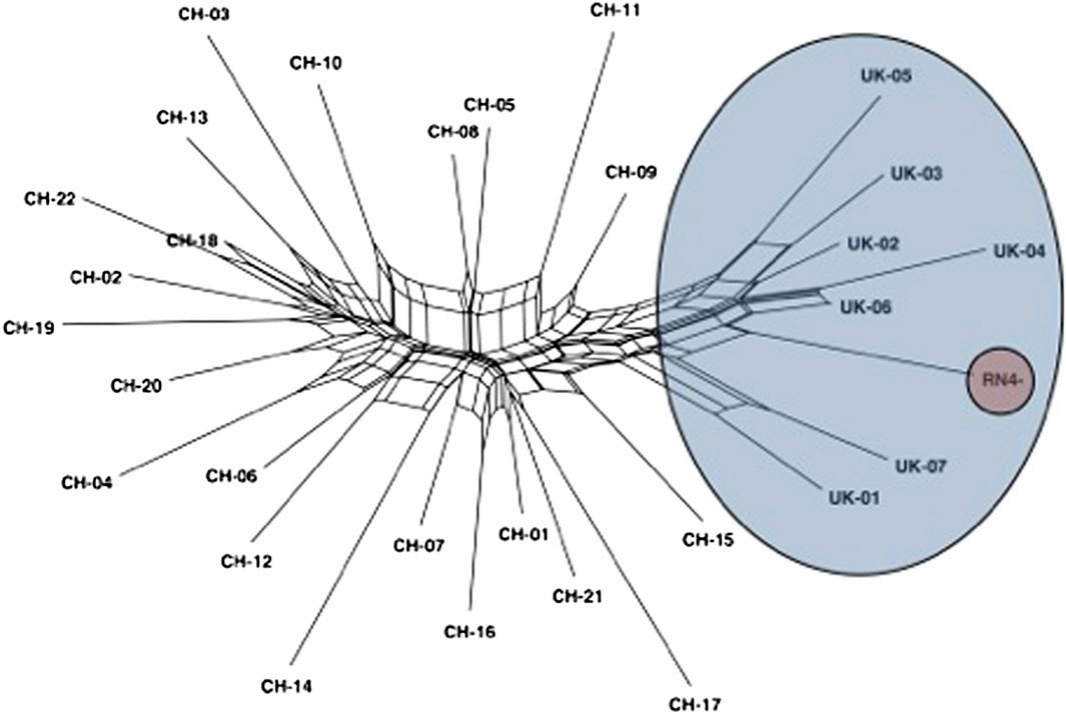
Neighbor network illustrating relationships among rats sampled from China (CH), the United Kingdom (UK), and the *Rattus norvegicus* reference genome (RN4). The network was estimated using SplitsTree4 (Huson and Bryant 2006). The UK samples are highlighted in blue and the reference in red.

We calculated population differentiation using Wright’s *F_st_* between the UK and China. Across all loci, *F_st_* = 0.254 (95% confidence [95% CI] 0.16−0.41, obtained by bootstrapping by locus), indicating a significant level of population differentiation. The China and UK samples come from populations showing subtle differences in morphology that have been classified into different subspecies (*caraco* and *norvegicus*, respectively), but the extent of gene flow between these populations is unknown. We estimated the neighbor network for all 29 samples and the orthologous regions from the reference genome using the program SplitsTree4 (Huson and Bryant 2006). The UK and Chinese samples cluster into two distinct groups, with a substantial amount of reticulation (parallel edges), indicating recombination among shared alleles (Figure 1). This finding is consistent with recent shared ancestry and/or ongoing gene flow. In addition, the genome reference sequence (RN4) is more genetically similar to the UK rats than the Chinese.

To further investigate population genetic structure, we used the software STRUCTURE v2.3.2 (Pritchard *et al.* 2000). We expected that STRUCTURE should capture additional information from patterns of linkage disequilibrium created by differentiation. To encode our sequence data as loci for STRUCTURE, we randomly drew a single SNP from each locus after removing singletons, which are uninformative. To ensure that our results were well supported, we bootstrapped the analysis 100 times, choosing a random SNP per locus for each replicate. For each replicate and K = 1 to 6 subpopulations, we ran five independent chains using the admixture with correlated allele frequencies model of STRUCTURE (1,000,000 iterations, burn-in of 200,000 iterations).

**Figure 2:**
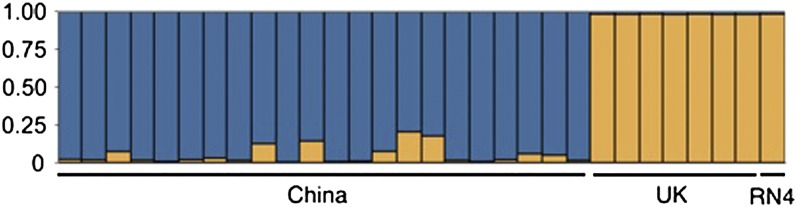
Genetic structure of *Rattus norvegicus* based on analyses conducted using STRUCTURE with *K* = 2 clusters shown in blue and orange. Each thin vertical bar represents an individual, who may be partitioned into *K* clusters depending on the estimated multilocus membership to each cluster. Each color represents the posterior probability of that individual belonging to a cluster. The measure Δ*K* from Evanno *et al.* (2005) indicates that *K* = 2 is the best-fitting model.

After the documentation we inferred the allele frequency parameter (λ) from the data for *K* = 1 and fixed λ for the subsequent runs (λ∼0.3). For each value of *K*, we averaged the inferred ancestry of each individual across all 100 bootstrap replicates and plotted these values in Figure 2. We also ran the same model of STRUCTURE with each haplotype at a locus encoded as an allele, after removing singletons. We found both methods of encoding the data gave similar conclusions. The results clearly support the existence of genetic differentiation between Chinese and UK rats, with no evidence of admixture (Figure 2). A number of methods have been proposed to determine the best-fitting model from a STRUCTURE analysis. Evanno *et al.* (2005) proposed using the maximum value of a statistic, Δ*K*, which is a function of the rate of change of posterior probability of the data, given the number of clusters, to identify the “true” number of clusters. Applying this method to our results with haplotypes encoded as alleles, the optimal number of clusters is two (Δ*K* = 627.6), where the Chinese and UK populations are clearly defined, and the genome reference sequence (RN4) clusters closely with UK rats (Figure 2). When haplotypes are encoded as alleles, RN4 forms a distinct subpopulation when *K* = 3, but this clustering is not supported in any of the bootstrap replicates (data not shown).

## Discussion

As expected, the RN4 reference strain is more similar to UK than Chinese rats because RN4 is believed to be derived from a wild-caught ancestor originating in Europe (Hedrich 2000). Based solely on the extent of shared alleles, RN4 is indistinguishable from wild UK rats (Figure 1). Similarly, STRUCTURE does not support significant genetic differentiation between RN4 and UK (Figure 2). We observed similar levels of nucleotide diversity in UK and China and significant genetic differentiation. Brown rats are believed to have colonized Europe from Asia only a few centuries ago, and rats can migrate for long distances via shipping. It is therefore possible that there is genetic subdivision within Asia and that there exists a population containing greater levels of diversity. Further sampling from other named subspecies of the brown rat, particularly in China [*socer* and *humiliatus* (Wu 1982)], might help to resolve this issue.

Having an estimate of autosomal nucleotide diversity (*θ*) in brown rats allows estimation of the recent effective population size (*N*_e_) of the species by equating silent site diversity to its equilibrium expectation under neutrality (4*N_e_μ*), where μ is the mutation rate per site. However, we observed marginally but not significantly negative Tajima’s *D*, so the brown rat population may not be at equilibrium, which could be explained by a recent population expansion (Fu and Li 1993). Alternatively, selective sweeps affecting coding sequences also generate an excess of rare alleles (Braverman *et al.* 1995), and a recent simulation study suggests that the predicted distortion of the site frequency spectrum is compatible with what is commonly observed in population samples of protein-coding loci (P. W. Messer and D. A. Petro, unpublished results). Because the allele frequency distribution does not allow discrimination between sweeps and population expansion (Przeworski 2002), we apply *θ* = 4*N_e_μ*, recognizing that we may underestimate the recent effective size if the population is expanding. This approach also includes the effect of purging of variation by selective sweeps or background selection, which increase the variance in genomic contributions. Comparative analysis of mammalian genomes suggests that nucleotide divergence down the rat lineage since the common ancestor with the house mouse is about 10–20% greater than down the mouse lineage (Rat Genome Sequencing Project Consortium 2004; Lindblad-Toh *et al.* 2011). Assuming that mice and rats diverged 12MYA (Benton and Donoghue 2007), that rats undergo two generations/year, and that synonymous divergence is 0.19 (Halligan *et al.* 2010), we can estimate *µ* = ∼4.2 × 10^−9^ per generation. Equating synonymous autosomal nucleotide diversity (0.0022; Table 1) to 4*N_e_μ* yields an estimate of *N_e_* in brown rats of 130,000. We calculated a multilocus, maximum likelihood (ML) estimate of *θ* given the number of segregating sites and samples following Wright *et al.* (2003). We estimated the 95% CI around ML *θ* using the χ^2^ approximation. ML *θ* = 0.0022, which was similar to our point estimate, and the 95% CI was 0.0014−0.0031. Assuming the same value for the mutation rate as used previously, the effective size of the rat population is between 85,000 and 184,000, which is much smaller than an estimate for the *M. m. castaneus* population from the species’ putative ancestral range in NW India (Halligan *et al.* 2010) but consistent with a negative relationship between population density (which is positively correlated with population size) and body size (Damuth 1981; Lynch 2007), and adult brown rats are typically about 20 times heavier than adult house mice.

## Supplementary Material

Supporting Information
