# Supplementary material for: Nuclear Gene Variation in Wild Brown Rats
Source: G3 (Bethesda). 2012 Dec 1;2(12):1661–4. doi: 10.1534/g3.112.004713 (PMC3516487; doi:10.1534/g3.112.004713)
Supplement: Supporting Information [file supp_2_12_1661__index.html]

Supporting Information 

# Nuclear Gene Variation in Wild Brown Rats

## Supporting Information for Ness *et al.*, 2012

**Files in this Data Supplement:**

- Supporting Information - Tables S1 and S2 (PDF, 100 KB)
- Table S1 - Table of locations of the 29 rats sampled for this study from China (CH) and the United Kingdom (UK) (PDF, 79 KB)
- Table S2 - Table of loci used in this study. Including their coordinates in the Rat reference genome (RN4), Gene ID, size and primers (PDF, 100 KB)
