# Supplementary material for: Nuclear Gene Variation in Wild Brown Rats
Source: G3 (Bethesda). 2012 Dec 1;2(12):1661–4. doi: 10.1534/g3.112.004713 (PMC3516487; doi:10.1534/g3.112.004713)
Supplement: Supporting Information [file supp_2.12.1661_004713SI.pdf]

Supplementary Table S1. Table of locations of the 29 rats sampled for this study from China (CH) and the United Kingdom (UK).

Sample ID	Country	Latitude	Longitude
CH01	China	45.8884	126.6291
CH02	China	45.6183	126.5849
CH03	China	45.8826	126.5691
CH04	China	45.6797	126.6097
CH05	China	45.6194	126.5869
CH06	China	45.8258	126.7839
CH07	China	45.8265	126.7839
CH08	China	45.8112	126.7688
CH09	China	45.6856	126.6347
CH10	China	45.6856	126.6329
CH11	China	45.6859	126.6379
CH12	China	45.6391	126.6678
CH13	China	45.6175	126.6749
CH14	China	45.8744	126.5711
CH15	China	45.8826	126.5698
CH16	China	45.8825	126.5684
CH17	China	45.6564	126.5931
CH18	China	45.6601	126.5996
CH19	China	45.6313	126.6160
CH20	China	45.6818	126.6159
CH21	China	45.6759	126.6146
CH22	China	45.6559	126.6008
UK01	United Kingdom	55.9429	-2.7934

UK02	United Kingdom	55.9438	-3.1810
UK03	United Kingdom	56.0260	-3.7914
UK04	United Kingdom	55.5870	-1.6784
UK05	United Kingdom	55.8593	-4.2410
UK06	United Kingdom	55.9321	-2.3607
UK07	United Kingdom	55.8691	-2.9728

Supplementary Table S2. Table of loci used in this study. Including their coordinates in the Rat reference genome (RN4), Gene ID, size and primers.

Locus ID	Ensembl Gene ID	Chromosome	RN4 Coords of Gene		Left Primer Coords	Right Primer Coords	Primer Product Size	PrimerF<br>PrimerR
chr_1A	ENSRNOG00000025400	1	180753943	180753411	180753945		180754475	530
chr_1B	ENSRNOG00000019309	1	182152992	182152441	182152992		182153543	551
chr_2A	ENSRNOG00000018488	2	148040390	148039733	148040421		148041048	627
chr_2B	ENSRNOG00000022625	2	213687099	213686582	213687102		213687602	500
chr_3A	ENSRNOG00000010054	3	158051358	158050795	158051370		158051922	552
chr_4A	ENSRNOG00000011808	4	84148687	84148047	84148695		84149287	592
chr_5A	ENSRNOG00000017577	5	149279682	149279137	149279698		149280222	524
chr_6A	ENSRNOG00000009491	6	42780020	42779490	42780033		42780551	518
chr_7A	ENSRNOG00000026916	7	10166609	10165710	10166680		10167476	796
chr_8A	ENSRNOG00000028360	8	20266352	20265666	20266378		20267024	646
chr_8B	ENSRNOG00000018467	8	65486515	65485758	65486557		65487271	714
chr_9A	ENSRNOG00000022822	9	83962763	83962253	83962768		83963271	503
chr_10A	ENSRNOG00000027770	10	90289908	90289298	90289910		90290452	542
chr_11A	ENSRNOG00000050701	11	17241547	17241022	17241550		17242066	516
chr_12A	ENSRNOG00000001961	12	22079134	22078629	22079136		22079636	500
chr_13A	ENSRNOG00000010113	13	88312987	88312306	88313007		88313657	650
chr_13B	ENSRNOG00000004319	13	98243546	98243027	98243546		98244063	517

chr_14A	ENSRNOG00000004153	14	20042681	20041856	20042717	20043496	779
chr_14B	ENSRNOG00000011338	14	87950754	87950222	87950775	87951287	512
chr_15A	ENSRNOG00000012300	15	3826966	3826341	3827068	3827579	511
chr_16A	ENSRNOG00000013291	16	6444261	6443746	6444264	6444764	500
chr_17A	ENSRNOG00000016992	17	96817220	96816641	96817250	96817784	534
chr_18A	ENSRNOG00000023433	18	77151516	77150736	77151530	77152289	759
chr_19A	ENSRNOG00000020859	19	49814030	49813487	49814040	49814562	522
chr_20A	ENSRNOG00000059548	20	5175027	5174427	5175028	5175606	578
chr_1C	ENSRNOG00000013945	1	82306314	82305178	82306646	82307408	762
chr_2C	ENSRNOG00000043292	2	42586965	42585838	42587155	42587929	774
chr_3B	ENSRNOG00000043045	3	52696860	52695718	52697161	52697956	795
chr_4B	ENSRNOG00000008870	4	173567443	173566199	173567623	173568429	806
chr_5B	ENSRNOG00000017523	5	167097250	167095890	167097348	167098198	850
chr_6B	ENSRNOG00000024310	6	40912533	40911055	40912757	40913528	771
chr_7B	ENSRNOG00000024998	7	87035949	87035023	87036105	87036867	762
chr_8C	ENSRNOG00000013171	8	111845569	111844732	111845635	111846398	763
chr_9B	ENSRNOG00000012663	9	45781694	45780154	45781958	45782752	794
chr_10B	ENSRNOG00000003217	10	108389551	108388459	108389700	108390551	851
chr_11B	ENSRNOG00000000716	11	2261687	2260590	2261726	2262526	800
chr_12B	ENSRNOG00000042213	12	9641141	9640315	9641191	9641941	750
chr_13C	ENSRNOG00000009691	13	45837708	45835522	45837827	45838897	1070

chr_14C	ENSRNOG00000003067	14	20042681	20041859	20042717	20043496	779
chr_15B	ENSRNOG00000037884	15	105090728	105089718	105090826	105091635	809
chr_16B	ENSRNOG00000014185	16	24921447	24919567	24922466	24923299	833
chr_17B	ENSRNOG00000018661	17	51250572	51249634	51250586	51251317	731
chr_18B	ENSRNOG00000019799	18	30717036	30714601	30717555	30718408	853
chr_19B	ENSRNOG00000039152	19	29215911	29212636	29217221	29217973	752
chr_20B	ENSRNOG00000000640	20	21886336	21885096	21886431	21887092	661
