# Supplementary material for: Nuclear Gene Variation in Wild Brown Rats
Source: G3 (Bethesda). 2012 Dec 1;2(12):1661–4. doi: 10.1534/g3.112.004713 (PMC3516487; doi:10.1534/g3.112.004713)
Supplement: Supporting Information [file supp_2.12.1661_TableS1.pdf]

Supplementary Table S1. Table of locations of the 29 rats sampled for this study from China (CH) and the United Kingdom (UK).

Sample ID	Country	Latitude	Longitude
CH01	China	45.8884	126.6291
CH02	China	45.6183	126.5849
CH03	China	45.8826	126.5691
CH04	China	45.6797	126.6097
CH05	China	45.6194	126.5869
CH06	China	45.8258	126.7839
CH07	China	45.8265	126.7839
CH08	China	45.8112	126.7688
CH09	China	45.6856	126.6347
CH10	China	45.6856	126.6329
CH11	China	45.6859	126.6379
CH12	China	45.6391	126.6678
CH13	China	45.6175	126.6749
CH14	China	45.8744	126.5711
CH15	China	45.8826	126.5698
CH16	China	45.8825	126.5684
CH17	China	45.6564	126.5931
CH18	China	45.6601	126.5996
CH19	China	45.6313	126.6160
CH20	China	45.6818	126.6159
CH21	China	45.6759	126.6146
CH22	China	45.6559	126.6008
UK01	United Kingdom	55.9429	-2.7934

UK02	United Kingdom	55.9438	-3.1810
UK03	United Kingdom	56.0260	-3.7914
UK04	United Kingdom	55.5870	-1.6784
UK05	United Kingdom	55.8593	-4.2410
UK06	United Kingdom	55.9321	-2.3607
UK07	United Kingdom	55.8691	-2.9728
